# High fatigue scores before and after surgical treatment of bone and soft tissue tumors

**DOI:** 10.3892/etm.2012.786

**Published:** 2012-10-31

**Authors:** INGRID C.M. VAN DER GEEST, HANS KNOOP, RENÉ P.H. VETH, H.W. BART SCHREUDER, GIJS BLEIJENBERG

**Affiliations:** 1 Department of Orthopaedic Surgery;; 2 Expert Centre of Chronic Fatigue, Radboud University Nijmegen Medical Centre, Nijmegen, The Netherlands

**Keywords:** bone tumor, soft tissue tumor, fatigue, quality of life

## Abstract

The first objective of the present study was to investigate fatigue severity in patients diagnosed with bone and soft tissue tumors prior to the surgical treatment of the tumor and 6 months post-operatively. The second objective was to determine which variables are associated with severe fatigue. Patients diagnosed with benign or low-grade malignant bone and soft tissue tumors, undergoing surgical therapy for the tumor only, were included in this study. The control group contained patients scheduled for knee arthroscopy for suspected meniscus tears. Fatigue, pain, anxiety and self-efficacy were measured pre-operatively and after 6 months and each patient wore an actometer to quantify physical activity. In the tumor group of 43 patients, 35% were severely fatigued pre-operatively and 33% post-operatively. The tumor group reported a significantly higher level of anxiety. No differences were observed in pain, physical limitations, self-efficacy or actometer scores. Multiple regression analysis of the tumor group revealed that higher pain scores, higher state anxiety and lower self-efficacy were asssociated with fatigue severity. In the control group of 24 knee arthroscopy patients, the percentage of severely fatigued patients decreased from 38% (n=9) prior to treatment to 29% (n=7) 6 months later. A substantial number of patients were severely fatigued in both the tumor group and the knee arthroscopy group. Self-efficacy, pain and anxiety appear to be the most important variables associated with fatigue severity in tumor patients prior to surgery.

## Introduction

In the majority of studies concerning cancer-associated fatigue, no clear association between fatigue and the type of cancer treatment has been revealed (1,2). Severe fatigue was not only observed in patients with malignant tumors but also in patients treated for benign bone tumors (3,4). Severe fatigue remained present in over a quarter of patients after a 2-year disease-free period and appears to be associated with tumor recurrence, less patient optimism, lower self-efficacy and increased somatization. It is unclear to what extent the fatigue already existed prior to the treatment of the tumor.

While fatigue is known to be an important factor influencing the quality of life of patients, little is known about the development of fatigue before, during or after tumor treatment and its contributing factors. Few studies have measured fatigue in patients before or during the treatment of a tumor (5–7).

Therefore, the first objective of the present study was to investigate fatigue severity in patients diagnosed with benign or low-grade malignant bone and soft tissue tumors prior to the surgical treatment of the tumor and 6 months post-operatively. The second objective was to determine which variables within the tumor group are significantly associated with fatigue severity. Variables which were expected to contribute to severe fatigue were selected, including physical limitations, physical (in)activity, pain, self-efficacy and anxiety. It was expected that decreased levels of physical activity caused by local pain from the tumor and weight-bearing restrictions, as well as the more physical limitations, would be associated with a higher level of fatigue (8). Anxiety was expected to be higher in the tumor group, both in benign and malignant tumors, due to the knowledge of having a tumor, the stress of the surgical treatment and the fear of local recurrence. A higher pain score and level of anxiety were expected to be associated with a higher level of fatigue. An association between higher self-efficacy and lower fatigue severity was demonstrated in a previous study (6).

The level of fatigue was assessed before the patients underwent surgery (the only tumor treatment) and 6 months post-operatively. Fatigue severity and possible associated factors in the tumor patients were compared with those of patients undergoing knee arthroscopy for suspected meniscus tears. The knee arthroscopy patients were selected as the control group, since these patients suffer from knee pain and decreased physical activity but do not have the psychological stress of a tumor diagnosis. The level of fatigue in the arthroscopy and tumor groups was also compared with published data from healthy controls.

## Patients and methods

### 

#### Patients

Consecutive patients recently diagnosed with benign or low-grade malignant bone and soft tissue tumors at the Department of Orthopaedic Surgery of the Radboud University Medical Centre Nijmegen (Nijmegen, The Netherlands) aged between 18 and 75 years and undergoing surgical therapy for the tumor only were asked to participate in the present study. Patients receiving chemotherapy or radiotherapy as adjuvant treatments were excluded from the study. Other exclusion criteria included previous treatment for cancer and severe co-morbidity causing fatigue. The control group contained patients undergoing knee arthroscopy for suspected meniscus tears, aged between 18 and 75 years, without severe co-morbidity causing fatigue. The tumor and the arthroscopy groups received the same assessment and this was performed prior to surgery and 6 months later. Permission for this study was given by the ethics committee of the medical centre. A written informed consent was signed by all participating patients.

### Assessment

#### Fatigue severity

Fatigue was measured with the fatigue severity subscale of the checklist individual strength (CIS) (2,9). This subscale contains 8 items scored on a 7-point Likert scale. Scores range between 8 (no fatigue) and 56 (severe fatigue). A score of ≥35 points indicates severe fatigue. This score is higher than the mean plus two standard deviations of a healthy control group (9,10). The CIS fatigue subscale has been used in studies concerning chronic fatigue syndrome and post-cancer fatigue and measures fatigue severity over the past 2 weeks. Since this questionnaire has a cut-off point for severe fatigue, it may be used to identify severe fatigue in individual patients and also for prevalence estimates in populations. Normative data are available for healthy controls. In the present study, the fatigue scores of the 2 patient groups were compared with the fatigue scores of a group of 53 healthy adults with a mean age of 37.1 years (SD, 11.5) (10).

#### Pain.

Current pain was measured with an 11-point numeric rating scale (NRS) with a score of 0 for no pain and 10 for severe pain. The pain subscale of the SF-36, in which patients have to indicate the magnitude of their pain symptoms and the impairment to daily functioning caused by this pain, was used to assess the impact of pain. Scores range between 0 and 100 (100 = no pain and no impairments due to pain; 0 = maximal pain and maximal impairment due to pain) (11,12).

#### Physical limitations.

Physical limitations were measured with the SF-36 subscale of physical functioning. This score ranges between 0 (very low physical functioning) and 100 (optimal physical functioning) (11,12).

#### Anxiety.

The level of anxiety was measured with the Spielberger State-Trait Anxiety Inventory version Y (STAI-form Y) (13,14). Only the subscale state anxiety was used, since the present level of anxiety was relevant to the present study. Scores range between 20 and 80 and a higher score reflects a higher level of anxiety.

#### General self-efficacy.

General self-efficacy, a general sense of personal competence and effectiveness, was measured with the general self-efficacy scale. The total score ranges between 10 and 40 and a higher score reflects a higher sense of control (15,16).

#### Physical activity.

An actometer (Actilog V3.0), a motion-sensing device worn at the ankle for 12 consecutive days and nights, was used to quantify physical activity. The actometer detects movements of the leg (e.g., during walking or climbing stairs). A general physical activity score that expressed the mean activity level over this period as the mean number of accelerations per 5-min intervals was calculated (17). Higher scores indicate a higher level of physical activity. Self-reported physical activity was measured on an 11-point NRS, with a score of 0 for ‘not physically active’ and 10 for ‘physically very active’.

#### Statistical analysis

Statistical analysis was performed using SPSS software (version 16.0). Descriptive statistics were used for description of the sample. The differences in fatigue, pain, physical activity, anxiety and self-efficacy between the tumor and arthroscopy group were tested using t-tests for independent samples. A t-test for independent samples was also used to compare the levels of fatigue of the 2 patient groups with a group of healthy controls. Pearson correlation analyses were used to determine the associations between fatigue severity and demographic, psychological and/or physical variables in the tumor group.

The variables that significantly correlated with fatigue severity were used as predictors in a multiple regression (backward method) with fatigue severity as the dependent variable. Only significant variables were entered in the multiple regression. As the sample size was expected to be rather small, this step was performed to limit the number of predictors. P<0.05 in the t-test and correlations were considered to indicate statistically significant differences. For the multiple regression, a P-value of 0.10 was used as the criterion for the removal of the variable from the regression. The significant predictors resulting from the regression analysis were considered to be possible determinants of fatigue.

## Results

### 

#### Patients

In the tumor patient group, 48 patients were included prior to the treatment of the tumor. At the 6-month follow-up, four patients refused to complete the assessment and were excluded from the analysis. One patient was also excluded due to a cerebrovascular accident during treatment. Of the remaining 43 patients, 20 were male (47%) and 23 female (53%). The mean age was 41.5 years (range, 19–67 years) and the tumors were located in the upper extremity (40%) or the lower extremity (60%). Treatment consisted of local excision (soft tissue tumors, osteochondroma) or curettage and cryosurgery (aneurysmal bone cysts, giant cell tumor, fibrous dysplasia, aggressive enchondroma, low-grade chondrosarcoma). One patient in the follow-up period underwent a second surgery to explore the previously operated area for suspected local recurrence but no local recurrence was identified. All other tumor patients were disease-free during the follow-up period. In the control group of knee arthroscopy patients, 24 patients completed the assessment before and 6 months after the arthroscopy. There were 13 males (54%) and 11 females (46%) in this group with a mean age of 43.1 years (range, 23–68 years). During the 6-month follow-up, 2 patients underwent a second surgery due to medial gonarthrosis, 1 patient underwent a high tibial osteotomy and 1 patient received an unicondylar knee prosthesis.

#### Fatigue severity in the tumor and knee arthroscopy group

In the tumor group, 35% of the patients were severely fatigued prior to treatment and 33% at the 6-month follow-up. The CIS fatigue score decreased significantly between pre- and post-operative assessment in the tumor and arthroscopy groups (Table I). The decrease over time in the CIS score was not significantly different between the tumor and arthroscopy groups [F (1.65)=0.326; P=0.570]. The mean CIS score at follow-up of the 2 groups was markedly higher than that of the healthy controls: 26.1 (SD, 13.7) in the tumor group and 24.4 (SD, 12.6) in the arthroscopy group, compared with a mean CIS-fatigue score of 17.3 (SD, 10.1) in the healthy control group (6).

In the arthroscopy group 9 patients (38%) were severely fatigued prior to the surgery. Of these 9 patients, four remained severely fatigued 6 months later without any physical problems associated with the surgery. The 3 knee arthroscopy patients that developed severe fatigue during or after treatment all underwent a second knee surgery or had persistent knee complaints. In the tumor patient group, only one patient underwent a second surgery and all patients were without evidence of disease 6 months after treatment.

#### Pain, physical limitations, physical activity, anxiety and self-efficacy in both patient groups

Scores for pain and anxiety decreased over time, whereas the actometer and physical functioning scores increased. No significant differences were observed between the tumor and arthroscopy groups with respect to the changes in these variables. The score for self-efficacy did not change over time as expected. Pre- and post-operative actometer scores were not compared, due to the small sample size of the pre-operative group (Table I).

#### Correlation of fatigue with other variables in the tumor patients

In the tumor group the NRS pain score, SF-36 pain, SF-36 physical functioning, anxiety and self-efficacy correlated significantly with fatigue severity (Table II). The anxiety score in the tumor patient group did not significantly differ between a benign and malignant diagnosis (t-test, P=0.405).

#### Multiple regression

Multiple regression analysis was used to identify predictors of severe fatigue in the tumor group 6 months after treatment (Table III). Factors significantly correlated with the CIS fatigue score in the previous study were entered in the analysis (anxiety at follow-up SF-36 pain at follow-up and self-efficacy at follow-up), as well as the CIS fatigue score before treatment.

Analysis revealed that higher CIS fatigue scores prior to treatment and higher state anxiety at follow-up were predictors of fatigue severity that explained ∼55% of the variance.

## Discussion

The primary objective of the present study was to determine the level of fatigue in patients diagnosed with benign and low-grade malignant bone and soft tissue tumors prior to the surgical treatment of the tumor and 6 months post-operatively. It was revealed that a substantial number of patients (38%) were severely fatigued. Six months after surgical treatment, 33% of the patients remained severely fatigued. When this group of severely fatigued patients was analyzed, it was apparent that the majority of the severely fatigued patients before treatment remained fatigued 6 months later. This severe fatigue cannot be explained by the physical condition of the patients, since all lacked evidence of disease and no complications from the surgical treatment occurred. Multiple regression analysis confirmed that a high CIS fatigue score prior to treatment was the most successful predictor of severe fatigue 6 months later. The CIS fatigue score decreased significantly over time but the percentage of patients severely fatigued before and after treatment remained similar.

In the knee arthroscopy group, severe fatigue was present to the same extent as in the tumor group prior to treatment. This supports the theory that severe fatigue is not induced by the tumor itself but by symptoms caused by the tumor or a meniscus tear, including pain, restricted physical activity and anxiety. In the arthroscopy group, severe fatigue at follow-up was associated with a second surgery or persistent knee complaints in approximately half of the patients.

It appears that severe fatigue is a symptom that develops prior to treatment and, if present at the clinical diagnosis, often persists over time. Interventions for persistent fatigue following cancer treatment, such as a physical training programs or cognitive behavioral therapy (18–20) have been demonstrated to be successful at reducing fatigue severity. With the results of the present study revealing that the majority of patients suffer from severe fatigue prior to treatment, we suggest that it may be useful to perform a screen for pre-existing fatigue severity prior to diagnosis and begin intervention for severe fatigue as early as possible after treatment.

## Figures and Tables

**Table I:** CIS fatigue, pain, anxiety, self-efficacy and physical functioning scores before and 6 months after surgical treatment.

	Tumor (n=43), mean (SD)			Arthroscopy (n=24), mean (SD)		
Characteristic	Pre-op	Follow-up	P-value	Pre-op	Follow-up	P-value
CIS fatigue	29.7 (14.2)	26.1 (13.7)	0.023	29.7 (13.1)	24.4 (12.6)	0.090
Pain (NRS)	2.7 (2.5)	2.1 (2.1)	0.061	2.3 (3.1)	1.8 (2.2)	0.445
SF-36 pain	57.0 (26.2)	71.8 (23.7)	0.000	66.5 (22.0)	82.8 (16.4)	0.007
SF-36 physical functioning	60.5 (26.8)	72.6 (23.7)	0.000	61.9 (20.7)	82.9 (18.7)	0.000
State anxiety	37.5 (10.5)	32.9 (10.1)	0.006	30.1 (7.7)	29.0 (9.9)	0.498
Self-efficacy	32.1 (4.5)	31.8 (4.8)	0.763	34.3 (4.3)	34.2 (4.3)	0.951

CIS, checklist individual strength; NRS, numeric rating scale; SD, standard deviation. Pre-op, pre-operatively.

**Table II:** Correlation of variables with fatigue severity (CIS fatigue)^a^ in tumor patients prior to surgical treatment.

Variable	Tumor (n=48)
Pain (NRS)	0.525^c^
SF-36 pain	−0.598^c^
SF-36 physical functioning	−0.338^b^
State anxiety	0.355^b^
Self-efficacy	−0.343^b^
Physical activity (self-reported NRS)	−0.215
Actometer	−0.026

a Pearson-r;

b P<0.05;

c P<0.01; CIS, checklist individual strength; NRS, numeric rating scale.

**Table III:** Results of multiple regression analysis to determine the contribution of variables to fatigue severity (CIS fatigue) in tumor patients 6 months after surgical treatment.

Predictor	B	Beta	P-value
Constant	−2.903		
State anxiety (follow-up)	0.401	0.302	0.033
CIS-fatigue (pre-op)	0.505	0.539	0.000
R^2^ adjusted	0.553		

CIS, checklist individual strength. Pre-op, pre-operatively. B, baseline, Beta, at follow-up.
